# Impaired health-related quality of life due to elevated risk of developing diabetes: A cross-sectional study in Indonesia

**DOI:** 10.1371/journal.pone.0295934

**Published:** 2023-12-20

**Authors:** M. Rifqi Rokhman, Bustanul Arifin, Benedetta Broggi, Anne-Fleur Verhaar, Zulkarnain Zulkarnain, Satibi Satibi, Dyah Aryani Perwitasari, Cornelis Boersma, Qi Cao, Maarten J. Postma, Jurjen van der Schans

**Affiliations:** 1 Department of Health Sciences, University of Groningen, University Medical Center Groningen, Groningen, The Netherlands; 2 Institute of Science in Healthy Ageing & HealthcaRE (SHARE), University Medical Center Groningen, University of Groningen, Groningen, The Netherlands; 3 Faculty of Pharmacy, Universitas Gadjah Mada, Yogyakarta, Indonesia; 4 Faculty of Pharmacy, Universitas Hasanuddin, Makassar, Indonesia; 5 Disease Prevention and Control Division, Banggai Laut Regency Health, Population Control and Family Planning Service, Central Sulawesi, Indonesia; 6 Unit of Pharmaco Therapy, Epidemiology and Economics (PTE2), Department of Pharmacy, University of Groningen, Groningen, The Netherlands; 7 Faculty of Medicine, Universitas Syiah Kuala, Banda Aceh, Indonesia; 8 Thyroid Center, Zainoel Abidin Hospital, Banda Aceh, Indonesia; 9 Faculty of Pharmacy, Universitas Ahmad Dahlan, Yogyakarta, Indonesia; 10 Faculty of Management Sciences, Open University, Heerlen, The Netherlands; 11 Department of Pharmacology and Therapy, Faculty of Medicine, Universitas Airlangga, Surabaya, Indonesia; 12 Department of Economics, Econometrics and Finance, Faculty of Economics & Business, University of Groningen, Groningen, The Netherlands; 13 Center of Excellence for Pharmaceutical Care Innovation, Universitas Padjadjaran, Bandung, Indonesia; Universitas Padjadjaran, INDONESIA

## Abstract

**Background:**

This study investigated the association between elevated risk of developing diabetes and impaired health-related quality of life (HRQoL) in the Indonesian population.

**Methods:**

A cross-sectional study was conducted on 1,336 Indonesians from the general population who had no previous diagnosis of diabetes. Utility score to represent HRQoL was measured using the EuroQol 5-dimension, while the risk for developing diabetes was determined using the Finnish Diabetes Risk Score (FINDRISC) instrument. All participants underwent a blood glucose test after fasting for 8 hours. The association between FINDRISC score and HRQoL adjusted for covariates was analysed using multivariate Tobit regression models. Minimal clinically important differences were used to facilitate interpretation of minimal changes in utility score that could be observed.

**Results:**

The median (interquartile range) of the overall FINDRISC score was 6 (7), while the mean (95% confidence intervals) of the EQ-5D utility score was 0.93 (0.93–0.94). Once adjusted for clinical parameters and socio-demographic characteristics, participants with a higher FINDRISC score showed a significantly lower HRQoL. No significant association was detected between fasting blood glucose level categories and HRQoL. A difference of 4–5 points in the FINDRISC score was considered to reflect meaningful change in HRQoL in clinical practice.

**Conclusion:**

An elevated risk of developing diabetes is associated with a lower HRQoL. Therefore, attention should be paid not only to patients who have already been diagnosed with diabetes, but also to members of the general population who demonstrate an elevated risk of developing diabetes. This approach will assist in preventing the onset of diabetes and any further deterioration of HRQoL in this segment of the Indonesian population.

## Introduction

The number of people living with diabetes worldwide is predicted to increase by 46% up from 537 million in 2021 to 783 million by 2045 [1]. As one of the most rapidly developing countries in Southeast Asia, Indonesia currently ranks fifth in the number of people with type 2 diabetes (T2D), with more than 19.4 million people with diabetes, and where the prevalence of diabetes in people aged 20–79 years has increased from 5.1% in 2011 to 10.8% in 2021 [1, 2]. The increase of diabetes diagnoses is further exacerbated by the fact that according to the International Diabetes Federation, there are still more than 14.3 million people living with diabetes undiagnosed in Indonesia [1].

In tandem with diabetes progression, health-related quality of life (HRQoL) is negatively affected and will reduce [3]. In its early developing stages, diabetes is characterized as an asymptomatic disease, making it difficult for early detection. Consequently, a substantial proportion of the population will have already experienced diabetes complications before a formal diagnosis of diabetes is made [4]. Although previous studies have reported that those with diabetes had significantly poorer HRQoL due to diabetes complications [5, 6], HRQoL had already diminished noticeably in individuals who had been detected as having impaired glucose tolerance [3]. Therefore, it is necessary to assess the HRQoL of individuals with normal glucose levels but who are at high-risk for developing diabetes in the future, before they actually develop impaired glucose tolerance.

Future risk for developing T2D can be assessed using a diabetes risk score by calculating known risk factors of diabetes [7]. This risk score is simple and non-invasive in predicting individuals at elevated risk for developing T2D. Since extending and enhancing a patient’s life and life quality is the utmost goal of patient care, HRQoL represents an additional data source for examining clinical outcomes [8, 9]. Effectively, even when HRQoL data cannot be obtained directly from an individual, just by estimating the risk of T2D based on a FINDRISC score alone may indirectly provide insight into a patient’s HRQoL. From a health economic evaluation perspective, information about the association of estimated risk for developing T2D and impaired HRQoL is crucial for assessing the burden of (pre)diabetes, and in evaluating the benefits of diabetes screening and prevention programs for reducing the burden. Diabetes prevention programs may not only assist to delay or prevent the occurrence of T2D [10], but also improve overall HRQoL to a clinically meaningful degree [11].

Three previous studies have evaluated the HRQoL in persons with elevated risk of developing diabetes [12–14]. These studies reported that people at high-risk for diabetes have a lower HRQoL in comparison with the general population in Finland, Norway, and Latin America. However, very limited studies concerning the link between the elevated risk for developing diabetes and HRQoL currently exist. Only one study in Finland looks at how low HRQoL is significantly associated with an estimated risk of developing T2D [15].

HRQoL is specific for each country, and no such studies regarding the association of diabetes risk and HRQoL exists for the Asian region. Therefore, this study investigated the association between the risk of developing T2D and HRQoL as adjusted by clinical parameters and socio-demographic characteristics relevant to the Indonesian population, as well as the association between blood glucose level categories and HRQoL.

## Materials and methods

### Study design

A cross-sectional study was carried out in Yogyakarta, Malang (East Java Province) and Banggai Laut Regency (Central Sulawesi Province). Data was collected through questionnaires, measurements of fasting blood glucose, height, weight, and waist circumference from June to November 2019. Three models were developed to investigate associations between risk of developing T2D, covariates, and HRQoL.

### Participants

Participants for the study were selected if they had never been diagnosed with type 1 or 2 diabetes mellitus, and were at least 18 years of age. Participants were excluded from the study if they were taking any medications that potentially have an effect on blood glucose (such as hydrochlorothiazide and beta-blockers) or if they had medical conditions that could affect blood glucose (such as anorexia).

In our study, we used a convenience sampling. Upon receiving ethical approval and permission from the Agency for National Unity and Politics to start our data collection, we contacted government authorities, such as village heads, neighbourhood heads, and head office of several institutions. We requested them to announce to the community or their employees that we would conduct our research. Prior to the study, all prospective participants were required to fast for a minimum of 8 hours before the assessment of their fasting blood glucose. We provided brief information of the research objectives, ethics, and procedures of the study to the prospective participants before they decided to join in our study. To avoid bias in measuring fasting blood glucose levels, participants who were not fasting for at least 8 hours were measured the following day.

Yogyakarta and Malang were categorized as Java area, while Banggai Laut Province was classified as Sulawesi area. For both Java and Sulawesi area, the minimum total sample was 385 participants in accordance with Daniel’s sample size formula to determine the sample size for cross-sectional study [16]. Parameters used to determine the minimum sample size were 95% for the level of confidence, 1.96 for the Z_1-α/2_ value, 0.5 for the expected proportion, and 0.05 for the absolute error or precision.

### Instruments

Study instruments consisted of socio-demographic questionnaires, Indonesian language versions of the FINDRISC and EQ-5D-5L, and clinical characteristic forms. Study instruments were completed by the participants, followed by measurements of participants’ waist circumference, body mass index (BMI) and fasting blood glucose levels by research assistants. Finally, the research assistants checked the study instruments of each participant for completion. Permission to use the FINDRISC instrument was obtained from the American Diabetes Association (ADA) as the copyright owner with document number KL111121-UOG, while permission to use EQ-5D-5L was obtained from the EuroQol research group with registration number 58203.

### Finnish Diabetes Risk Score (FINDRISC)

The risk of developing T2D was estimated using the FINDRISC instrument. At its conception, FINDRISC was initially developed to detect individuals at future risk of having T2D, but in recent years, the instrument has also been used to identify people with prediabetes and undiagnosed T2D [7, 17]. FINDRISC was originally developed for English-speaking populations, but it has since been translated, adapted and validated for Asian populations [18–20]. The Indonesian version of this instrument is a preferable diagnostic tool with a good predictive accuracy [21]. The diagnostic accuracy of this Indonesian version indicated by the area under the curve is 0.73 (0.67–0.78) for screening undiagnosed T2D, and 0.72 (0.69–0.75) for detecting undiagnosed T2D and prediabetes [21]. FINDRISC items and related scoring can be found in S1 Table.

Eight items related to T2D risk factors were included in FINDRISC instrument; namely age, BMI, waist circumference, daily physical activity, fruits and vegetables consumption, use of blood pressure medications, a medical history of high blood glucose, and a family history of diabetes [7]. Each item consists of several answer choices and each with various weighted scores. The FINDRISC total score can range between 0 and 26, of which a higher score indicates a greater risk for developing diabetes.

The FINDRISC score was categorized in accordance with the original FINDRISC study for developing T2D, and the risk categories comprise of low (<7), slightly elevated (7–11), medium (12–14), high (15–20) and very high (>20). In this study, a modification was made by combining participants in the high-risk and very high-risk categories since only 11 of the 1,336 participants were classified as belonging to the very high-risk category.

### Health-related quality of life (HRQoL)

HRQoL was estimated using the EuroQol 5-dimension 5-level instrument (EQ-5D-5L). This instrument provides a single, overall health utility score that facilitates the estimation of quality-adjusted life years (QALYs) for health technology assessments. The EQ-5D-5L instrument is a standardized generic instrument to assess HRQoL, and consists of 2 elements [22, 23].

The first element is the descriptive system of EQ-5D-5L that covers 5 dimensions (namely mobility, self-care, usual activities, pain or discomfort, and anxiety or depression), where each dimension has 5 levels of options, ranging from no problems to extreme problems [23]. The second element is the EuroQol Visual Analogue Scale (EQ-VAS) that ranges between 0 (for the worst health state a participants can imagine) and 100 (for the perfect health state). The EQ-5D utility score of a specific health state in the descriptive system can be estimated by combining each level of the five dimensions using a value set, while the EQ-VAS utility score is derived by dividing the number marked on the scale by 100. Both utility scores that calculated from the descriptive system and EQ-VAS ranging from 0 to 1. Participants with a higher utility score represent those with a higher or better quality of life.

Minimally important differences can be used to facilitate interpretation of minimal changes in the EQ-5D utility score that can be observed in an individual and that are meaningful in clinical practice [24, 25]. Thus, in this study, a distribution-based approach was used, and an equation of 0.2*standard deviation is proposed to calculate minimally important differences [25].

### Covariates

The relationship between FINDRISC score and HRQoL when full covariates were included was calculated with socio-demographic characteristics and clinical parameters as control variables. Socio-demographic data included gender, age, educational level, and study location. We categorized Yogyakarta and Malang as Java. Compared to the Banggai Laut Regency, that is located in a remote area, Malang and Yogyakarta had some similarities in terms of geographical location, accessibility, and health facilities.

Clinical parameters of participants included BMI, waist circumference, the number of classical diabetes symptoms, and fasting blood glucose level. The weight, height and waist circumference of each participant were measured by qualified research assistants using standard procedures. BMI and waist circumference characteristics were also included upon recommendation by WHO classifications [26, 27]. BMI was classified into 4 groups: underweight (BMI <18.5 kg/m^2^), normal (BMI 18.5-<25 kg/m^2^), overweight (BMI 25–30 kg/m^2^), and obese (BMI >30 kg/m^2^); while waist circumference was divided into 3 categories based on gender and pursuant to metabolic complication risk: for women (normal <80 cm, increased risk >80–88 cm, substantially increased risk >88 cm), while for men (normal <94 cm, increased risk >94–102 cm, substantially increased risk >102 cm). Classical diabetes symptoms, and the corresponding numbers, were determined by assessing the presence of polyuria, polyphagia, polydipsia, slow-healing wounds, and blurred vision. Participants were asked whether they experienced blurred vision symptom within the last six months specifically and the last three months for the other classical symptoms of diabetes, of polyuria, polyphagia, polydipsia, and slow-healing wounds.

The measurement of fasting blood glucose was conducted using a finger-prick test (EasyTouch®GCU) administered by a physician or nurse. Participants were then divided into 3 categories in which as having normal fasting blood glucose (<100 mg/dL), prediabetes (100–125 mg/dL) or undiagnosed T2D (>126 mg/dL) following guidelines set by the Indonesian Society of Endocrinology [28].

### Statistical methods

EQ-5D utility scores were generated using the Indonesian value set [29], and EQ-VAS utility scores were also calculated. The FINDRISC score was reported by using median and interquartile range (IQR), while EQ-5D utility scores were reported using mean and 95% confidence intervals. EQ-5D utility scores were calculated for each group categorized under clinical parameters and sociodemographic characteristics. Mann-Whitney tests were applied to compare EQ-5D utility scores between two groups and not normally distributed, while Kruskal Wallis tests were performed to compare EQ-5D utility scores of more than two groups.

Logistic regression model was not applied as we did not use dichotomous outcome variables (e.g., yes/no) as our outcomes but HRQoL scores. Also, multivariate linear regression models could not be used since the data was too negatively skewed and not normally distributed, while also an ordinal regression model by dividing the HRQoL based on the percentile values also could not be performed since the 60^th^ and 80^th^ of percentiles had the same values. In addition, our data had a ceiling effect where 54.3% of participants had perfect health status and EQ-5D utility scores = 1. A Tobit regression model was therefore considered to be the most appropriate approach for our specific dataset to analyse the association between the FINDRISC and HRQoL. The Tobit regression model is used since regression methods can produce biased estimates by not taking into account for the ceiling effect in measuring the health status [30]. Some previous studies especially when analysing the HRQoL with a ceiling effect applied this regression model [31, 32]. All FINDRISC items and HRQoL were first assessed and, subsequently, six models were developed to examine associations between FINDRISC scores, socio-demographic characteristics, clinical parameters, and HRQoL.

In the first model, associations between FINDRISC scores and EQ-5D utility scores were investigated in an unadjusted model, while clinical parameters relating to fasting blood glucose categories were added to the second model. Finally, other clinical parameters and socio-demographic characteristics were added to create a third, most comprehensive model to investigate how the associations changed when adjusting for socio-demographic characteristics (gender, age, educational level, and study location) and other clinical parameters (BMI, waist circumference, the number of classical diabetes symptoms). Associations between FINDRISC scores and EQ-VAS utility scores were also evaluated according to these 3 models. The coefficients for each regression model were displayed together with 95% of confidence intervals. Pseudo r-square values were calculated for each model, and it usually has positive values ranging between 0 and 1. However, in the Tobit regression, the pseudo r-square can also be negative if both values of the constant-only and full model log-likelihoods are positive [33].

We used complete-case analysis since only 4.8% of total respondents with missing values of their HRQoL. A two-tailed *p-*value of less than .05 was defined as a statistically significant association. Univariate associations were investigated using the Statistical Package for Social Sciences (SPSS) version 26.0, while STATA Special Edition 16.1 was used for all regression analyses.

### Ethical approval

The research objectives, ethics, and procedures were explained to all potential participants. All individuals participated in the study provided their written informed consent (S1 File). Ethical approval was acquired from the Ethics Committee of the Faculty of Dentistry of the Universitas Gadjah Mada Yogyakarta, Indonesia on 25 April 2019 as set out in approval notice 0095. All participants were given health supplements, in particular, iron supplement and multivitamins. Additional information regarding the ethical, cultural, and scientific considerations specific to inclusivity in global research is included in S1 Checklist.

## Results

### Characteristics of study participants

In total, 1,336 out of 1,403 (95.2%) participants completed the questionnaires (S1 Dataset). Two participants (0.2%) were excluded due to negative HRQoL while 65 participants (4.6%) did not have data regarding HRQoL. There were 532 participants (39.8%) from Yogyakarta, 242 participants (18.1%) from Malang, and 562 participants (42.1%) from Banggai Laut. The distribution of participants based on socio-demographic characteristics, clinical parameters, and FINDRISC categories is presented in Table 1. The overall median (IQR) of the FINDRISC score was 6 (7). The number of participants classified to each FINDRISC category was significantly different and, in general, higher FINDRISC categories contained substantially more participants that were women, of older age, a higher BMI and waist circumference, and those who had a higher number of classical diabetes symptoms. In addition, the median (IQR) of fasting blood glucose levels of total participants was 92 mg/dL (IQR = 17). In the categories with a higher FINDRISC score, the median (IQR) fasting blood glucose levels and the percentage of participants with prediabetes and undiagnosed diabetes was significantly higher.

**Table 1:** Distribution of participants in FINDRISC-Indonesian version (n = 1,336).

Characteristic	Group	Total (%)	FINDRISC category (%)				*p*-value
			Low risk	Slightly Elevated risk	Moderate risk	High risk	
Total participants		100.0	50.3	32.4	9.3	7.9	-
*Socio-demographic characteristics*							
Gender	Women	61.2	54.0	68.6	66.4	74.2	< .001
	Men	38.8	46.0	31.4	33.6	25.8	
Age	<45 years	61.8	74.5	53.9	36.9	36.6	< .001
	45–54 years	16.5	13.3	17.8	25.4	22.6	
	55–64 years	11.0	6.8	14.5	18.9	16.1	
	>64 years	10.7	5.5	13.8	18.9	24.7	
Education level	Up to Junior high school	18.3	15.6	19.4	29.5	19.4	< .001
	Senior high school	29.0	31.4	25.1	29.5	28.0	
	Undergraduate	47.9	50.1	48.5	36.1	44.1	
	Postgraduate	4.8	2.9	7.0	4.9	8.6	
Study location	Java	57.9	52.9	63.5	64.8	61.3	.002
	Sulawesi	42.1	47.1	36.5	35.2	38.7	
*Clinical parameters*							
Body mass index	Underweight (<18.5 kg/m^2^)	7.3	12.2	2.3	1.6	1.1	< .001
	Normal (18.5-<25 kg/m^2^)	50.9	67.0	38.6	32.0	11.8	
	Overweight (25-<30 kg/m^2^)	31.3	20.5	43.6	41.8	41.9	
	Obese (>30 kg/m^2^)	10.5	0.3	15.5	24.6	45.2	
Waist circumference	Men <94 cm; women <80 cm	48.2	74.8	22.0	20.5	6.5	< .001
	Men >94–102 cm; women >80–88 cm	25.1	18.2	34.4	32.8	24.7	
	Men >102 cm; women >88 cm	26.6	7.1	43.6	46.7	68.8	
Classical diabetes symptoms	No symptoms	47.8	51.1	46.5	43.1	35.5	< .001
	1 symptom	28.9	29.4	30.2	23.6	25.8	
	2 symptoms	14.5	13.1	14.0	20.3	20.4	
	3 symptoms	6.1	4.5	7.2	6.5	12.9	
	>4 symptoms	2.3	1.9	1.4	5.7	5.4	
Fasting blood glucose	Normal (<100 mg/dL)	70.8	83.9	63.7	47.5	36.6	< .001
	Prediabetes (100–125 mg/dL)	23.4	13.5	30.4	40.2	43.0	
	Undiagnosed diabetes (>126 mg/dL)	5.8	2.6	5.9	12.3	20.4	
Fasting blood glucose, median (IQR)		92 (17)	89 (15)	95 (18)	101 (21)	104 (22)	< .001
FINDRISC score, median (IQR)		6 (7)	3 (4)	9 (2)	13 (1)	16 (3)	< .001

*Notes*. FINDRISC = Finnish Diabetes Risk Score; IQR = interquartile range;

*p-value < .01;

**p-value < .001.

**Fig 1:**
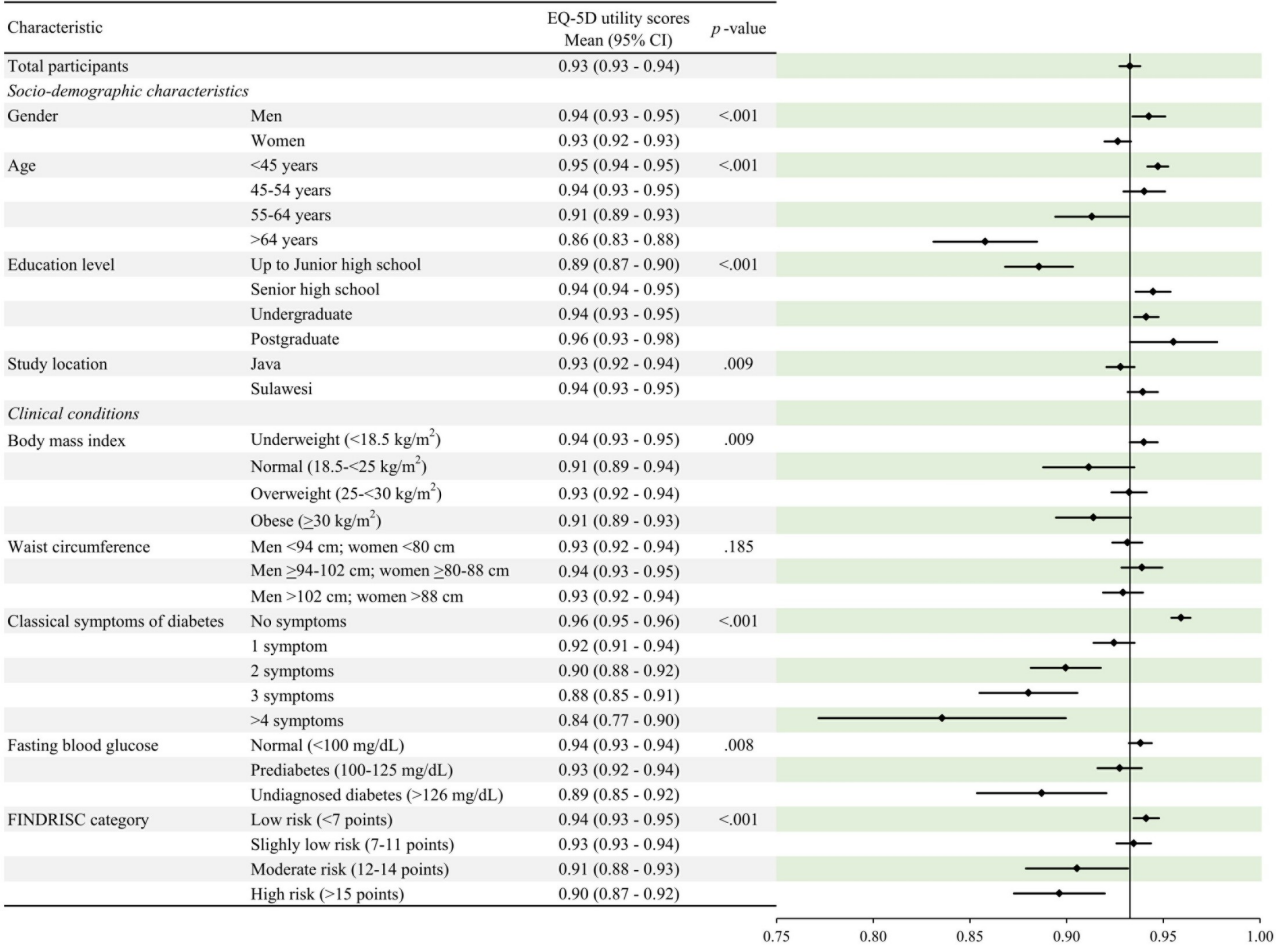
Distribution of participants on EQ-5D utility score (n = 1,336). *Notes*. 95% CI: 95% confidence interval.

The distribution of the EQ-5D utility scores is shown in Fig 1, and the overall utility score was determined to be 0.93 (0.93–0.94). The HRQoL was significantly lower in women participants (*p* < .001), older (*p* < .001), with higher BMI (*p* = .009), higher numbers of diabetes symptoms (*p* < .001), higher fasting blood glucose levels (*p* < .008), lower formal education levels (*p* < .001), and who resided in Java (*p* = .009). The lowest HRQoL at 0.84 (0.77–0.90) was experienced by participants with the highest number of classical diabetes symptoms, while the highest HRQoL at 0.96 (0.93–0.98) was found in postgraduate participants.

### The association between FINDRISC components and HRQoL

The association between FINDRISC components and EQ-5D utility scores is presented in Table 2. All components of FINDRISC had a negative association with EQ-5D utility scores, except for waist circumference (Table 2), which was not significantly associated (*p*-values > .05). FINDRISC items that were significantly associated with EQ-5D utility scores consisted of age (*p* < .001 for participants in 55–64 and >64 age groups), BMI (*p* = .012 for participants with BMI >30 kg/m^2^), fruits and vegetables consumption (*p* = .001), and a history of high blood glucose (*p* = .006). Age had the highest negative coefficient, followed by BMI, history of high blood glucose, and fruits and vegetables consumption.

**Table 2:** Association between FINDRISC components and EQ-5D utility scores using multivariate Tobit regression (n = 1,336).

FINDRISC components	Coefficient	95% CI		*p*-value
		Lower	Upper	
Age				
< 45 years	Ref.			
45–54 years	-0.03	-0.06	<0.01	.053
55–64 years	-0.08	-0.12	-0.05	< .001***
>64 years	-0.15	-0.18	-0.11	< .001***
Body mass index				
<25 kg/m^2^	Ref.			
25–30 kg/m^2^	-0.02	-0.04	0.01	.259
>30 kg/m^2^	-0.06	-0.10	-0.01	.012*
Waist circumference				
Men <94 cm; women <80 cm	Ref.			
Men >94–102 cm; women >80–88 cm	0.02	-0.01	0.05	.099
Men >102 cm; women >88 cm	0.03	-0.01	0.06	.081
Physical activity (30 min/day)				
Yes	Ref.			
No	-0.02	-0.05	0.01	.095
Fruit and vegetable consumption				
Every day	Ref.			
Not every day	-0.04	-0.06	-0.02	.001**
Antihypertensive medication				
No	Ref.			
Yes	-0.03	-0.06	0.01	.080
History of high blood glucose				
No	Ref.			
Yes	-0.05	-0.09	-0.02	.006**
Family members with diabetes				
No	Ref.			
Grandparent, aunt, uncle, or cousin	-0.02	-0.06	0.01	.177
Parent, brother, sister, or child	0.01	-0.02	0.04	.397

*Notes*. FINDRISC = Finnish Diabetes Risk Score; Ref: reference;

*p-value < .05;

**p-value < .01;

***p-value < .001.

### The association between FINDRISC score, fasting blood glucose category, and HRQoL

The association between FINDRISC score and EQ-5D utility score is as shown in Table 3. In all three models, FINDRISC scores had significant negative associations with EQ-5D utility scores. Adding socio-demographic characteristics and clinical parameters to the model increased pseudo r-square from 0.05 to 0.33, but only slightly lowered the coefficient of the FINDRISC scores from 0.006 to 0.005. In contrast, the fasting blood glucose category only correlated significantly in Model 2 (*p* = 0.041 for participants with undiagnosed T2D), but the addition of other clinical parameters and socio-demographic characteristics to the model diminished the correlation to the level of insignificance.

**Table 3:** Association between FINDRISC score, clinical conditions, sociodemographic characteristics, and EQ-5D utility score using multivariate Tobit regression analysis (n = 1,336).

Independent variable	Model 1	Model 2	Model 3
	pseudo r-square: 0.05	pseudo r-square: 0.05	pseudo r-square: 0.33
FINDRISC score	-0.006 (-0.009, -0.004)***	-0.006 (-0.008, -0.003)***	-0.005 (-0.008, -0.002)**
*Clinical parameters*			
Fasting blood glucose (vs Normal (<100 mg/dL))			
Prediabetes (100–125 mg/dL)		0.003 (-0.024, 0.030)	0.011 (-0.014, 0.036)
Undiagnosed diabetes (>126 mg/dL)		-0.049 (-0.096, -0.002)*	0.004 (-0.041, 0.048)
Body mass index (vs Normal (18.5-<25 kg/m^2^))			
Underweight (<18.5 kg/m^2^)			-0.029 (-0.069, 0.011)
Overweight (25-<30 kg/m^2^)			-0.027 (-0.054, >-0.001)*
Obese (>30 kg/m^2^)			-0.061 (-0.105, -0.017)**
Waist circumference (vs Men <94 cm; women <80 cm)			
Men >94–102 cm; women >80–88 cm			0.047 (0.017, 0.077)**
Men >102 cm; women >88 cm			0.074 (0.037, 0.112)***
Classical diabetes symptoms (vs No symptoms)			
1 symptom			-0.056 (-0.080, -0.032)***
2 symptoms			-0.085 (-0.115, -0.054)***
3 symptoms			-0.108 (-0.150, -0.066)***
>4 symptoms			-0.137 (-0.202, -0.073)***
*Socio-demographic characteristics*			
Women (vs Men)			-0.044 (-0.068, -0.020)***
Age (vs <45 years)			
45–54 years			-0.007 (-0.039, 0.025)
55–64 years			-0.041 (-0.078, -0.003)*
>64 years			-0.086 (-0.124, -0.047)***
Education (vs Up to Junior high school)			
Senior high school			0.051 (0.020, 0.082)**
Undergraduate			0.032 (<0.001, 0.064)*
Postgraduate			0.068 (0.013, 0.124)*
Sulawesi (vs Java)			0.005 (-0.023, 0.033)

*Notes*. FINDRISC = Finnish Diabetes Risk Score;

*p-value < .05;

**p-value < .01;

***p-value < .001.

In Model 3, aside from presenting a high FINDRISC score, participants with the lowest EQ-5D utility scores were women, of older age, lower education levels, a higher BMI, lower waist circumferences, and a higher number of classical diabetes symptoms. All variables reduced EQ-5D utility scores except two, namely education levels and waist circumference, which had a positive association. Two variables, namely fasting blood glucose level and living in Java or Sulawesi islands, did not significantly affect HRQoL. The variable with the highest coefficient in negative association with HRQoL was the number of classical diabetes symptoms, which had a coefficient range of 0.056 to 0.137.

The multivariate association between overall FINDRISC scores and EQ-5D utility scores, using a Tobit regression model, showed that the coefficient of the regression model was -0.0047 (rounded to 0.005 in Model 3) with a standard deviation in the utility scores of 0.10. Therefore, using a minimally important difference of 0.2*standard deviations of the utility score (0.2*0.10 = 0.02) divided by the coefficient, a discrepancy of 4–5 points in the FINDRISC score was estimated to provide meaningful changes in quality of life.

Table 4 displays negative associations between FINDRISC scores and EQ-VAS in all 3 models. In Model 3, besides FINDRISC score, a significant lower of EQ-VAS was experienced by women participants with underweight, higher education levels, having 3 classical symptoms of diabetes, and participants from Java.

**Table 4:** Association between FINDRISC score, clinical conditions, sociodemographic characteristics, and EQ-VAS using multivariate Tobit regression analysis (n = 1,336).

Independent variable	Model 1	Model 2	Model 3
	pseudo r-square: -0.024	pseudo r-square: -0.032	pseudo r-square: -0.275
FINDRISC score	-0.002 (-0.004, -0.001)**	-0.003 (-0.005, -0.001)**	-0.003 (-0.005, >-0.001)*
*Clinical parameters*			
Fasting blood glucose (vs Normal (<100 mg/dL))			
Prediabetes (100–125 mg/dL)		0.016 (-0.004, 0.036)	0.018 (-0.002, 0.038)
Undiagnosed diabetes (>126 mg/dL)		0.012 (-0.024, 0.048)	0.013 (-0.023, 0.049)
Body mass index (vs Normal (18.5-<25 kg/m^2^))			
Underweight (<18.5 kg/m^2^)			-0.048 (-0.080, -0.017)**
Overweight (25-<30 kg/m^2^)			-0.012 (-0.033, 0.009)
Obese (>30 kg/m2)			-0.011 (-0.045, 0.024)
Waist circumference (vs Men <94 cm; women <80 cm)			
Men >94–102 cm; women >80–88 cm			0.020 (-0.003, 0.043)
Men >102 cm; women >88 cm			0.015 (-0.014, 0.045)
Classical diabetes symptoms (vs No symptoms)			
1 symptom			0.011 (-0.008, 0.029)
2 symptoms			-0.009 (-0.033, 0.015)
3 symptoms			-0.052 (-0.086, -0.017)**
>4 symptoms			-0.020 (-0.074, 0.033)
*Socio-demographic characteristics*			
Women (vs Men)			-0.025 (-0.044, -0.007)**
Age (vs <45 years)			
45–54 years			0.009 (-0.016, 0.034)
55–64 years			-0.013 (-0.042, 0.017)
>64 years			-0.003 (-0.034, 0.029)
Education (vs Up to Junior high school)			
Senior high school			-0.026 (-0.051, -0.001)*
Undergraduate			-0.062 (-0.088, -0.037)***
Postgraduate			-0.065 (-0.106, -0.023)**
Sulawesi (vs Java)			0.063 (0.041, 0.085)***

*Notes*. FINDRISC = Finnish Diabetes Risk Score;

*p-value < .05;

**p-value < .01;

***p-value < .001.

## Discussion

This is the first population-based study that examined the association between estimated diabetes risk and impaired HRQoL in an Asian population. A negative association was discovered between FINDRISC scores and HRQoL. This negative association was still significant after clinical parameters and socio-demographic characteristics adjustment. This main finding is confirmed by another study that reported a high risk of diabetes led to a lower HRQoL in Finland [12, 15]. In our study, FINDRISC components that were found to be significantly associated with the quality of life comprised of age, BMI, fruits and vegetables consumption, and a history of high blood glucose.

Since FINDRISC scores had a negative association with EQ-5D utility scores, lowering FINDRISC scores can likely increase HRQoL. FINDRISC contains modifiable risk factors in terms of BMI, waist circumference, daily physical activity, and consumption of fruits and vegetables [7]. These modifiable risk factors are related to each other and can be reduced by implementing public health strategies or promoting lifestyle interventions. A previous meta-analysis showed that dietary and physical activity interventions in South Asians resulted in significant reduction in weight, waist circumference, and diabetes incidence [34], while other studies reported that reduction in weight and waist circumference increased the HRQoL [35–37]. Thus, implementing dietary and physical activity interventions as public health strategies will potentially reduce BMI and waist circumference, and increase the HRQoL. Generally, waist circumference has an inverse relationship with the HRQoL; however, in our study, only this variable that positively related to HRQoL. This unique relationship is in accordance with other previous studies that reported higher waist circumference is also a sign of prosperity and better mental health, and when higher waist circumference starts to impair physical function, the HRQoL will begin negatively correlated with waist circumference [38, 39].

Our findings are also corroborated by another study that showed a reduction of 4–5 points in FINDRISC score was estimated to provide meaningful improvements, or noticeable changes observed by health care professionals, in the quality of life of individual participants [15]. Notably, while Väätäinen et al. (2016) used a model without covariates to estimate minimally important differences, our study used a model with an adjustment for covariates. Without a covariate adjustment in our study, a difference of 3–4 points in a FINDRISC score would have been estimated to provide noticeable changes in quality of life. This estimation could be used by healthcare professionals in daily practice to inform and encourage participants of diabetes risk-reduction programs.

Participants with the lowest EQ-5D utility scores were women, older participants, with lower education levels, higher BMI, lower waist circumference, and having a higher number of classical symptoms of diabetes. Together with information about risk of developing diabetes, this information is valuable to formulate the characteristics of target population that might have greatest benefits of intervention to minimize the potential loss of HRQOL and early detection of high-risk patients before they develop impaired glucose tolerance. Previous study shows that subjective health can be improved and risk of T2D and its consequences may be reduced by regular exercise [12]. In our study, individuals with undiagnosed T2D had a utility score of 0.89 (0.85–0.92), and this utility score was higher than the utility score of patients with diabetes at 0.77 (0.75–0.79) reported in the previous study [6]. In our study, compared to individuals with undiagnosed T2D, individuals with normoglycaemia and prediabetes also had higher utility scores at 0.94 (0.93–0.94) and 0.93 (0.92–0.94), respectively. This indicates that the quality of life is decreasing over time along with the progression of diabetes.

Not all predictors associated with lower EQ-5D utility scores were significantly associated with EQ-VAS, namely age, waist circumference, and higher body mass index. This can be the case since EQ-VAS is more sensitive to anchor-point bias when participants interpret the worst or best imaginable health state differently, and as a result, they assign different values for similar health states [40, 41] In addition, EQ-VAS reflects more patient perspectives about how good or bad their health, while EQ-5D utility score is more represents the societal perspective and preferable from a health economic point of view [24].

Higher fasting blood glucose levels were not directly associated with HRQoL when other clinical parameters and socio-demographic characteristics were added to the model. Another study conducted in patients with diabetes in Indonesia also obtained similar findings that random, fasting, and post prandial blood glucose levels did not significantly affect HRQoL [6]. In our model, it is the diabetes-related symptoms variable that has the biggest impact on HRQoL. The early stages of diabetes, commonly referred to as prediabetes, is indicated by a long period and asymptomatic condition of elevated blood glucose levels [42]. This condition causes no complaints, and people with an elevated blood glucose level do not realize they are at risk of developing diabetes. As the condition develops further, the presence of diabetes-related symptoms will significantly lower HRQoL and, unfortunately, only people with pronounced diabetes-related symptoms or complications will seek help from a healthcare provider. This point highlights the importance of screening programs to detect onset or early stages of diabetes and to prevent deterioration in quality of life.

The strength of this study is the fact that data was collected from three regions across two islands in Indonesia which collectively represent both big cities (Yogyakarta, Malang) and a remote area (Banggai Laut Regency). These three regions were selected due to a notably higher prevalence of community diabetes compared to the national average [43]. so naturally it was predicted that these regions would also see a greater number of people at higher risk for developing diabetes. Moreover, participants in this study covered an extensive range of backgrounds, in term of ages and formal education levels. Concerning age, 61.8% of participants was less than 45 years, 16.5% between 45–54 years, 11.0% between 55–64 years, and 10.7% more than 64 years. These percentages were similar with the general proportions of Indonesia population based on data from the Central Agency on Statistics in 2019 in which 59.0% of population was between 20–45 years, 18.9% between 45–54 years, 12.9% between 55–64 years, and 9.2% more than 64 years [44].

Admittedly, some limitations of the study must be acknowledged. Firstly, the study applied a cross-sectional study design that cannot be used to determine causal relationships, and it lacks the ability to track diabetes risk and HRQoL over time. Further studies, using longitudinal study designs, are necessary to reveal the effects of modifiable risk factor intervention for diabetes such as the maintenance of a healthy diet or a lowering of BMI on a participant’s HRQoL. Secondly, as Indonesia is not genetically homologous, but rather home to an abundance of Asian ethnicities and ethnic subgroupings, further research to reveal the relationship between diabetes risk and ethnicity is required. Finally, minimally important differences were estimated using a distribution-based approach. Further studies that use a combined approach of a distribution and anchor-based methods are required. This combination method offers the advantages from both anchor-based method by comparing the meaningful changes in HRQoL to external criteria and the benefit from distribution-based method by offering higher stability across different sample [45]. However, this method would require a longitudinal study design with repeated measurements and external criteria as a reference, both of which were not available to us.

## Conclusions

Elevated risk for developing diabetes is associated with lower HRQoL, thus individuals with higher FINDRISC score are likely to have lower HRQoL. No association was detected between fasting blood glucose levels and HRQoL. Lower EQ-5D utility scores were also predominantly associated with being older, women, of a lower educational level, a higher BMI, and having a higher number of diabetes symptoms. Therefore, attention should be placed not only on patients with a diabetes diagnosis but also on members of the general population with an elevated risk of developing diabetes to prevent further disease progression and deterioration of HRQoL in such individuals.

## Supporting information

S1 Dataset (XLSX)

S1 Table Modified FINDRISC- Bahasa Indonesia instrument. (DOCX)

S1 File Research protocol. (DOCX)

S1 Checklist Inclusivity in global research. (DOCX)

S2 Checklist STROBE statement—checklist of items that should be included in reports of observational studies. (DOCX)
