# Supplementary material for: Impaired health-related quality of life due to elevated risk of developing diabetes: A cross-sectional study in Indonesia
Source: PLoS One. 2023 Dec 20;18(12):e0295934. doi: 10.1371/journal.pone.0295934 (PMC10732360; doi:10.1371/journal.pone.0295934)
Supplement: S1 File — (DOCX) [file pone.0295934.s003.docx]

**S1 File. Research protocol.**

- 1. **Research Subject**

Subjects for validation must meet the inclusion and exclusion criteria. The inclusion criteria are as follows: individuals who have never been diagnosed with diabetes mellitus (both types 1 and 2), 18 years old or older, fasting for at least 8 hours, and willing to sign a research informed consent after receiving an explanation about the study. The exclusion criteria are as follows: individuals taking drugs that could affect blood glucose levels (i.e. thiazides, beta blockers, and steroids) or participants with diseases or clinical conditions that affect blood glucose levels (i.e. anorexia nervosa, hepatitis, and pancreatic tumors). The number of participants in this study is 1,000 participants.

- 1. **Tools and instruments**

The tools used are:

1. GCU meter of Easy Touch brand. This tool can be used to measure blood sugar, cholesterol, and uric acid levels. In this research, the GCU meter is used to measure blood glucose level.

2. Gluco Strip of Easy Touch brand, which is a strip to measure participants' blood sugar and is used with GCU Meter.

3. Blood lancet

4. Alkohol swabs of One Med brand

5. Waist gauge

6. Stadiometer

The instruments needed are:

1. FINRISC-Bahasa Indonesia

2. Participants' sociodemographic data

3. EQ-5D-5L questionnaire

- 1. **Method**

1. Translation and Validation of FINDRISC

FINDRIS-English Version cannot be translated literally to the Indonesian Language. Cultural adaptation is required in the translation to maintain the meaning and purpose of the questions. Therefore, translation, pilot-testing, and validation are required. The validation permit for FINDRISC has been given by Prof. Jaakko Tuomilehto as the developer of the instrument and the American Diabetes Association (ADA) as the copyright holder.

1. Translation

The translation consists of 2 stages, namely: forward and backward translations. In the forward translation, the English version of FINRISC (the original version) will be translated into Indonesian Language by 2 professional translators working separately. The two translators are native speakers of Indonesian who work as a professional English translator (fluent in both Indonesian and English). The researcher will compare the translated instruments from the two translators. Minor differences can be resolved through the consensus of the two translators. However, if significant differences are found, a third translator must translate the instrument and be involved in discussions with the two translators to find a solution. The result of the forward translation, which is FINDRISC-Bahasa Indonesia, is called Version 1.

In the backward translation, FINDRISC Version 1 from the forward translation will be translated from Indonesian Language to English by 2 professional translators working separately. The two professional translators are native speakers of English who are fluent in Indonesian Language. Backward translation is performed to compare the original FINDRISC with the translated version and assess whether the meanings of the questions are similar to those of the translated version. If differences are found, the researcher will have discussions with the two translators. The final result of the translation process is FINDRISC-Bahasa Indonesia Version 2.

1. Pilot-testing

In the pilot-testing stage, interviews were done with 10-15 participants to determine whether or not there are questions which are difficult to answer, confusing or difficult to understand^2^. In this research, FINDRISC-Bahasa Indonesia Version 2 will be used in 3 groups of participants. The first group comprises 10 individuals with different age characteristics from the general population of Yogyakarta (age <45, 45-55, 55-65, and >65 years). The second group consists of 10 individuals with different age characteristics from the general population of Sulawesi (age <45, 45-55, 55-65, and >65 years). The third group comprises 10 individuals having at least a master's degree and the experience of translating an instrument or having the experience of conducting research using one of health instruments in Indonesia. If the participants agree, the interview process will be recorded. FINRISC-Bahasa Indonesia Version 2 was then revised according to feedback from the participants. The result of this stage is the final version of FINDRISC-Bahasa Indonesia which will be used for the validation process.

1. Validation

The validation involves 1000 participants.

Figure 1. The translation, pilot-testing and validation of FINRISC-English Version to FINRISC-Bahasa Indonesia

FINDRISC original version

Professional translator 1

Professional translator 2

Group 2

Version 2

Professional translator

English native speaker 1

Professional translator

English native speaker 2

Compared by researchers Peneliti

Compared by the researchers

Group 1

Version 1

Group 3

FINDRISC-Bahasa Indonesia

Participants

Translated by

Translated by

Forward translation

Pilot-testing

Validation

Backward translation

1. Filling Procedure of FINRISC-Bahasa Indonesia and Patient Examination

The research procedures and information will be explained to the participants. After the patients sign the informed consent, the participants will be asked to:

1. fill out the patient's sociodemographic questionnaire and the EQ-5D-5L
2. fill out the paper-based FINDRISC questionnaire with the help of a research assistant. The research assistants will measure body mass index by measuring the weight and height of the participants. The research assistants will also ask the participants to measure their waist circumference.
3. The research assistant will measure the fasting blood sugar of the participants. The procedure for measuring the fasting blood sugar is as follows:
4. The patient has fasted for at least 8 hours.
5. Take the green chip (blood sugar) and put it in the device, wait until it is "OK".
6. A number/code will appear on the screen according to the strip bottle.
7. After that, a picture of a blood drop will appear.
8. Insert the needle into the lancet/pen-shaped gun and adjust the depth of the needle according to the thickness of the participant’s skin (usually on number 3 or 4).
9. Use alcohol swabs to clean fingertips.
10. Shoot the needle on the fingertip of the left hand and press it so that the blood comes out.
11. Blood is touched on the side edge of the strip (not dripped over the center of the EasyTouch blood test strip).
12. Touch the line where there is an arrow and the blood will immediately seep to the end of the strip and a “beep” will sound.
13. Wait a moment, the results will appear for a few seconds on the screen.
14. Remove the needle from the lancet and collect it for destruction. One needle is only used for one participant.
15. The chip is stored in the bottle again.
16. Close the strip bottle tightly when not in use.

After filling out the FINDRISC-Bahasa Indonesia and completing the patient's fasting blood sugar check, the research assistant will help calculate the risk score of the participants and provide an explanation of the risk score.

- 1. **Data Analysis and Processing**

Data analysis is carried out by:

1. In the analysis of the characteristics of the research sample, tabulation is done with proportions (percentages) for categorical variables, and calculations of the mean and standard deviation (SD) for continuous variables are performed.

2. Logistic regression is used to evaluate the predictive ability of each risk factor in the FINDRISC score.

3. The discriminatory ability of FINDRISC to predict the development of type 2 diabetes is analyzed using the area under the receiver-operating curve (AUC-ROC). ROC is created by combining the 1-specificity value as the x value and sensitivity as the Y value.

4. EQ-5D-5L will be calculated with the value set for the Indonesian population.
