# Supplementary material for: Impaired health-related quality of life due to elevated risk of developing diabetes: A cross-sectional study in Indonesia
Source: PLoS One. 2023 Dec 20;18(12):e0295934. doi: 10.1371/journal.pone.0295934 (PMC10732360; doi:10.1371/journal.pone.0295934)
Supplement: S2 Checklist — (DOCX) [file pone.0295934.s005.docx]

STROBE Statement—checklist of items that should be included in reports of observational studies

|  | | Item No. | | Recommendation | Page No. | Relevant text from manuscript | | |
| --- | --- | --- | --- | --- | --- | --- | --- | --- |
| **Title and abstract** | | 1 | | (*a*) Indicate the study’s design with a commonly used term in the title or the abstract | Page 1 | Lines 1-2 | | |
|  |  |  |  | (*b*) Provide in the abstract an informative and balanced summary of what was done and what was found | Page 2 | Abstract, lines 52-70 | | |
| Introduction | | | | | |  | | |
| Background/rationale | | 2 | | Explain the scientific background and rationale for the investigation being reported | Page 3 | ‘Introduction’ section, lines 80-101 | | |
| Objectives | | 3 | | State specific objectives, including any prespecified hypotheses | Page 4 | ‘Introduction’ section, lines 102-105 | | |
| Methods | | | | | |  | | |
| Study design | | 4 | | Present key elements of study design early in the paper | Page 4 | ‘Material and Methods’ section, lines 108-112 | | |
| Setting | | 5 | | Describe the setting, locations, and relevant dates, including periods of recruitment, exposure, follow-up, and data collection | Page 4 | ‘Material and Methods’ section, lines 108-122 | | |
| Participants | | 6 | | (*a*) *Cohort study*—Give the eligibility criteria, and the sources and methods of selection of participants. Describe methods of follow-up  *Case-control study*—Give the eligibility criteria, and the sources and methods of case ascertainment and control selection. Give the rationale for the choice of cases and controls  *Cross-sectional study*—Give the eligibility criteria, and the sources and methods of selection of participants | Page 4 | ‘Material and Methods’ section, lines 114-122 | | |
|  |  |  |  | (*b*) *Cohort study*—For matched studies, give matching criteria and number of exposed and unexposed  *Case-control study*—For matched studies, give matching criteria and the number of controls per case | n/a |  | | |
| Variables | | 7 | | Clearly define all outcomes, exposures, predictors, potential confounders, and effect modifiers. Give diagnostic criteria, if applicable | Page 5-6 | ‘Material and Methods’ section, lines 134-185 | | |
| Data sources/ measurement | | 8* | | For each variable of interest, give sources of data and details of methods of assessment (measurement). Describe comparability of assessment methods if there is more than one group | Page 5-6 | ‘Material and Methods’ section lines 118-185 | | |
| Bias | | 9 | | Describe any efforts to address potential sources of bias | Page 4, 7 | ‘Material and Methods’ section, lines 118-122, lines 193-205 | | |
| Study size | | 10 | | Explain how the study size was arrived at | Page 4 | ‘Material and Methods’ section, lines 123-125 | | |
| Quantitative variables | 11 | | Explain how quantitative variables were handled in the analyses. If applicable, describe which groupings were chosen and why | | Page 5-6 | | ‘Material and Methods’ section, lines 147-150, 172-185 |  |
| Statistical methods | 12 | | (*a*) Describe all statistical methods, including those used to control for confounding | | Page 7 | | ‘Material and Methods’ section, lines 199-205 |  |
|  |  |  | (*b*) Describe any methods used to examine subgroups and interactions | | n/a | |  |  |
|  |  |  | (*c*) Explain how missing data were addressed | | Page 7 | | ‘Material and Methods’ section, lines 206 |  |
|  |  |  | (*d*) *Cohort study*—If applicable, explain how loss to follow-up was addressed  *Case-control study*—If applicable, explain how matching of cases and controls was addressed  *Cross-sectional study*—If applicable, describe analytical methods taking account of sampling strategy | | Page 7 | | ‘Material and Methods’ section, line 204-205 |  |
|  |  |  | (*e*) Describe any sensitivity analyses | | n/a | |  |  |
| Results | | | | | | | |  |
| Participants | 13* | | (a) Report numbers of individuals at each stage of study—eg numbers potentially eligible, examined for eligibility, confirmed eligible, included in the study, completing follow-up, and analysed | | Page 8 | | ‘Results’ section, lines 217-218 |  |
|  |  |  | (b) Give reasons for non-participation at each stage | | Page 8 | | ‘Results’ section, lines 217-218 |  |
|  |  |  | (c) Consider use of a flow diagram | | n/a | |  |  |
| Descriptive data | 14* | | (a) Give characteristics of study participants (eg demographic, clinical, social) and information on exposures and potential confounders | | Page 9 | | ‘Results’ section, Table 1 |  |
|  |  |  | (b) Indicate number of participants with missing data for each variable of interest | | Page 8 | | ‘Results’ section, lines 217-218 |  |
|  |  |  | (c) *Cohort study*—Summarise follow-up time (eg, average and total amount) | | n/a | |  |  |
| Outcome data | 15* | | *Cohort study*—Report numbers of outcome events or summary measures over time | | n/a | |  |  |
|  |  |  | *Case-control study—*Report numbers in each exposure category, or summary measures of exposure | | n/a | |  |  |
|  |  |  | *Cross-sectional study—*Report numbers of outcome events or summary measures | | Page 8 | | ‘Results’ section, line 233, Figure 1 |  |
| Main results | 16 | | (*a*) Give unadjusted estimates and, if applicable, confounder-adjusted estimates and their precision (eg, 95% confidence interval). Make clear which confounders were adjusted for and why they were included | | Page 12 | | ‘Results’ section, Table 3 (including 95% confidence interval) |  |
|  |  |  | (*b*) Report category boundaries when continuous variables were categorized | | Page 5-6 | | Lines 147-150, 172-185 |  |
|  |  |  | (*c*) If relevant, consider translating estimates of relative risk into absolute risk for a meaningful time period | | n/a | |  |  |

Continued on next page

| Other analyses | 17 | Report other analyses done—eg analyses of subgroups and interactions, and sensitivity analyses | n/a |  |
| --- | --- | --- | --- | --- |
| Discussion | | | | |
| Key results | 18 | Summarise key results with reference to study objectives | Page 13 | ‘Discussion’ section, lines 274-279 |
| Limitations | 19 | Discuss limitations of the study, taking into account sources of potential bias or imprecision. Discuss both direction and magnitude of any potential bias | Page 14 | ‘Discussion’ section, lines 312-322 |
| Interpretation | 20 | Give a cautious overall interpretation of results considering objectives, limitations, multiplicity of analyses, results from similar studies, and other relevant evidence | Page 13-14 | ‘Discussion’ section |
| Generalisability | 21 | Discuss the generalisability (external validity) of the study results | Page 14 | ‘Discussion’ section, lines 316-317 |
| Other information | |  | | |
| Funding | 22 | Give the source of funding and the role of the funders for the present study and, if applicable, for the original study on which the present article is based | Page 1 | Cover letter, paragraph 3 |

*Give information separately for cases and controls in case-control studies and, if applicable, for exposed and unexposed groups in cohort and cross-sectional studies.

**Note:** An Explanation and Elaboration article discusses each checklist item and gives methodological background and published examples of transparent reporting. The STROBE checklist is best used in conjunction with this article (freely available on the Web sites of PLoS Medicine at http://www.plosmedicine.org/, Annals of Internal Medicine at http://www.annals.org/, and Epidemiology at http://www.epidem.com/). Information on the STROBE Initiative is available at www.strobe-statement.org.
